# Editorial: Community series in unveiling the tumor microenvironment by machine learning to develop new immunotherapeutic strategies, volume II

**DOI:** 10.3389/fimmu.2024.1351597

**Published:** 2024-01-24

**Authors:** Ping Zheng, Jun Liu

**Affiliations:** ^1^ Department of Neurosurgery, Pudong New Area People’s Hospital, Shanghai, China; ^2^ Department of Medicine, Yuebei People’s Hospital, Shaoguan, China

**Keywords:** tumor microenvironment, machine learning, tumor, disease, immune infiltrations

A total of 25 papers are included in this series. We selected seven as representative:

Yang et al. discussed the role of lipid metabolism in gastric cancer. Their study identified 78 genes related to fatty acid metabolism that are differentially expressed between normal and gastric cancer tissues. The ConsensusClusterPlus R package was used to divide the genes into two gastric cancer subtypes, cluster 1 and cluster 2. Patients in cluster 2 were found to have a poorer prognosis than those in cluster 1. The study used machine learning to select 8 differentially expressed genes between the subtypes to construct a fatty acid prognostic risk score (FARS) model, which displayed good prognostic efficacy. Certain anticancer drugs, such as bortezomib, elesclomol, GW843682X, and nilotinib, showed significant sensitivity in the high FARS score group. RGS2 was identified as the core gene in gastric cancer single-cell analysis, and Western blotting and immunofluorescence staining results revealed high levels of expression of this gene in gastric cancer cells. The results of immunohistochemical staining showed that a large amount of RGS2 was deposited in the stroma in gastric cancer. The pan-cancer analysis also revealed a significant association of RGS2 with TMB, TIDE, and CD8+ T-cell infiltration in other cancer types as well. RGS2 may thus be further studied as a new target for immunotherapy in future studies on gastric cancer. The FARS model developed here enhances our understanding of lipid metabolism in the TME in gastric cancer, and provides a theoretical basis for predicting tumor prognosis and clinical treatment.

Peng et al. investigated the development of a prognostic model based on oxidative stress for lung adenocarcinoma (LUAD). The study extracted oxidative stress-related genes (ORGs) from Genecards and performed machine learning algorithms to build the OS-score and OS-signature. The study identified ten ORGs with prognostic value and the OS-signature containing three prognostic ORGs. The efficiency and accuracy of the OS-signature in predicting the prognosis for LUAD patients was confirmed by survival ROC curves and two external validation data sets. Patients with high OS-scores were found to have lower levels of immunomodulators, stromal score, immune score, ESTIMATE score, and infiltrating immune cell populations. Conversely, patients with higher OS-scores were more likely to have higher tumor purity. PCR assays showed that MRPL44 and CYCS were significantly upregulated in LUAD cell lines, while CAT was significantly downregulated.

Wang et al. discussed the role of MYBL1 in clear cell renal cell carcinoma (ccRCC). The study comprehensively investigated the role of MYBL1 in ccRCC and found that MYBL1 was correlated with progressive clinical characteristics and worse prognosis performance. The study also found that MYBL1 can activate multiple oncogenic pathways in ccRCC and can remodel the immune microenvironment of ccRCC and affect the immunotherapy response. *In vitro* and *in vivo* assays indicated that MYBL1 is upregulated in ccRCC cells and can promote the cellular malignant behavior of ccRCC. Finally, a machine learning algorithm - LASSO logistic regression was utilized to identify a prognostic signature based on the MYBL1-derived molecules, which showed satisfactory ability to predict patient prognosis in both training and validation cohorts. The study concluded that MYBL1 is a novel biomarker of ccRCC that can remodel the tumor microenvironment, influence immunotherapy responses, and guide precision medicine in ccRCC.

Ke et al. discussed the potential and significance of immune-related diagnostic biomarkers in differentiating Uterine leiomyosarcoma (ULMS) from Uterine leiomyoma (ULM). The study downloaded two public gene expression profiles containing ULMS and ULM samples and identified differentially expressed genes (DEGs) among 37 ULMS and 25 ULM control samples. The DEGs were used for Gene Ontology (GO), Kyoto Encyclopaedia of Genes and Genomes (KEGG), and Disease Ontology (DO) enrichment analysis in addition to gene set enrichment analysis (GSEA). The study identified DPP6 and MFAP5 as diagnostic biomarkers for ULMS, which were verified in the GSE9511 and GSE68295 datasets. Low expression of DPP6 and MFAP5 was associated with ULMS. The study concluded that DPP6 and MFAP5 are potential diagnostic biomarkers for ULMS.

Zhang et al. investigated the development of a mitochondria-related signature in osteosarcoma patients. Transcriptomic data and clinical information of osteosarcoma samples were collected from the Therapeutically Applicable Research to Generate Effective Treatments (TARGET) and Gene Expression Omnibus (GEO) databases. Comprehensive bioinformatics analysis was performed on the samples at the bulk RNA sequencing level and single-cell RNA sequencing (scRNA-seq). The study constructed a mitochondria-related signature in osteosarcoma patients and explored its prognostic value, predictive value in the immune microenvironment and chemotherapeutic agents. The study also investigated the association between mitochondria and immunity in the tumor microenvironment of osteosarcoma at the scRNA-seq level. The tumorigenic role of the critical mitochondria-related gene, PCCB, was verified by *in vitro* validation. The study concluded that a mitochondria-related signature was developed in osteosarcoma with solid predictive values for the immune microenvironment, chemotherapeutic agents, and prognosis.

Xu et al. discussed the identification of a glycolysis and cholesterol synthesis-related genes (GCSRGs) signature for effective prognostic assessment of osteosarcoma patients. Gene expression data and clinical information were obtained from the GSE21257 and TARGET-OS datasets. Patients diagnosed with osteosarcoma were classified into one of 4 subtypes (quiescent, glycolysis, cholesterol, and mixed subtypes), which differed significantly in terms of prognosis and tumor microenvironment. Both univariate and LASSO Cox regression analyses were conducted on the screened module genes to identify 5 GCSRGs (RPS28, MCAM, EN1, TRAM2, and VEGFA) that constituted a prognostic signature for osteosarcoma patients. The signature was an effective prognostic predictor, independent of clinical characteristics, as further verified via Kaplan-Meier analysis, ROC curve analysis, and univariate and multivariate Cox regression analysis. Additionally, the GCSRG signature had a strong correlation with drug sensitivity, immune checkpoints and immune cell infiltration.

Cholangiocarcinoma (CHOL) is a prevalent type of malignancy and the second most common form of primary liver cancer, resulting in high rates of morbidity and mortality. Necroptosis is a type of regulated cell death that appears to be involved in the regulation of several aspects of cancer biology, including tumorigenesis, metastasis, and cancer immunity. Xu et al. aimed to construct a necroptosis-related gene (NRG) signature to investigate the prognosis of CHOL patients using an integrated bioinformatics analysis. CHOL patient data were obtained from the GEO (GSE89748, GSE107943) and TCGA databases, and NRG data from necroptosis were obtained from the KEGG database. A total of 65 differentially expressed (DE) NRGs were screened, of which five were selected to establish the prognostic signature of NRGs based on multivariate Cox regression analysis. Low-risk patients survived significantly longer than high-risk patients. Patients with high-risk scores experienced higher immune cell infiltration, drug resistance, and more somatic mutations than patients with low-risk scores. Sensitivities to GW843682X, mitomycin C, rapamycin, and S-trityl-L-cysteine were significantly higher in the low-risk group than in the high-risk group. Finally, the expression of five NRGs was validated.

All of the above studies select a specific geneset and identify prognosis-related or stage-related genes using machine learning methods, some of them further associate these genes with immune status and finally validate them with *in vitro* or *in vivo* experimental methods.

## Author contributions

PZ: Conceptualization, Data curation, Writing – original draft, Writing – review & editing. JL: Data curation, Investigation, Methodology, Writing – review & editing.

